# Middle East and North Africa Registry to Characterize Rate of *RAS* Testing Status in Newly Diagnosed Patients with Metastatic Colorectal Cancer

**DOI:** 10.5152/tjg.2022.22106

**Published:** 2023-02-01

**Authors:** Mohammed Oukkal, Kamel Bouzid, Adda Bounedjar, Abdulsalam Alnajar, Fouad Abou Taleb, Abdullah Alsharm, Hassen Mahfouf, Blaha Larbaoui, Amr Abdelaziz, Assia Ouamer, Linah Bashir

**Affiliations:** 1 Department of Medical Oncology, University Center of Beni Messous, Algiers, Algeria; 2 Department of Medical Oncology, Center Pierre & Marie Curie, Algiers, Algeria; 3 Department of Medical Oncology, University Center of Blida, Blida, Algeria; 4 GI Oncology Department, Kuwait Cancer Control Center, Sabah Health Region, Kuwait; 5 Department of Medical Oncology, Zagazig University Faculty of Medicine, Zagazih, Al-Sharkeyya, Egypt; 6 Department of Medical Oncology, King Fahad Medical City Hospital, Comprehensive Cancer Center, Riyadh, Kingdom of Saudi Arabia; 7 Department of Medical Oncology, Public Hospital of Rouiba, Algiers, Algeria; 8 Department of Medical Oncology, Cancer Center of Oran, Messerghine, Algeria; 9 Department of Medical Oncology, Alexandria University Faculty of Medicine, Alexandria, Egypt; 10 Department of Oncology, AMGEN Maghreb, Algiers, Algeria; 11 Department of Oncology, Hematology & Nephrology, AMGEN (Middle East), Dubai, United Arab Emirates

**Keywords:** Colorectal cancer, metastatic disease, observational trial, RAS testing

## Abstract

**Background:**

Rat sarcoma virus mutational status guides first-line treatment in metastatic colorectal cancer. This study was a multicenter, multi-country ambispective, observational study in the Middle East and North Africa assessing regional rat sarcoma virus testing practices in newly diagnosed patients.

**Methods:**

The retrospective arm (2011-2014) included adults with metastatic colorectal cancer who had initiated first-line therapy with ≥1 post-baseline visit and survival data. The prospective arm (2014-2019) enrolled newly diagnosed patients with histologically proven metastatic colorectal cancer with ≥1 measurable lesion per Response Evaluation Criteria in Solid Tumors, and tissue availability for biomarker analysis. Data look-back and follow-up were 2 years; the rate of *RAS* mutation was evaluated.

**Results:**

*RAS* testing was ordered for patients in retrospective (326/417) and prospective (407/500) studies. In the former, testing was typically prescribed after first-line treatment initiation, significantly more in patients with stage IV disease (*P *< .005), resulting in the addition of targeted therapy (41.8% anti-epidermal growth factor receptor, 30.2% anti-vascular endothelial growth factor) in wild-type metastatic colorectal cancer, and significantly impacted the treatment of left-sided tumors (*P *= .037). In the latter, 58.4% were *RAS* wild-type; 41.6% were *RAS* mutant. Non-prescription of *RAS* testing was attributed to test unavailability, financial, or medical reasons; predictors of testing prescription were older age, primary tumor in ascending colon, and high tumor grade. *RAS* status knowledge resulted in the addition of anti-vascular endothelial growth factor (20.4%) or anti-epidermal growth factor receptor therapy (21.2%).

**Conclusion:**

Before 2014, *RAS* testing in patients with colorectal cancer in the Middle East and North Africa was often performed after first-line treatment. Testing is more routine in newly diagnosed patients, potentially shifting early treatment patterns.

**Main Points**

- Data on rat sarcoma virus (*RAS*) status of metastatic colorectal cancer (mCRC) remain limited in the Middle Eastern and African regions.
- *RAS* status assessment is essential for decision-making in mCRC.
- *RAS *testing can significantly impact treatment decisions.

## Introduction

The incidence of colorectal cancer (CRC) is increasing worldwide. In 2018, nearly 1.8 million new cases of CRC were diagnosed, and more than 880 000 deaths were ascribed to CRC.^1^ The burden of CRC appears to be greater in developed countries, with an incidence estimated to be 3-fold higher when compared to developing countries. In the last decade, it has been reported that the incidence of CRC in the Arab world increased approximately 2- to 3-fold.^2^ Between 1998 and 2007, it was the second most common malignancy in the Gulf Cooperation Council (GCC) countries, accounting for nearly 8% of all cancers there. Kuwait has the highest incidence of CRC in this region, with 15.5 cases per 100 000 individuals.^2^ Of particular concern was the increasing trend observed among individuals under the age of 40.^2^ In Egypt, Algeria, and Lebanon, the age-standardized rates of CRC range from 4.9 to 16.3 per 100 000.^3-5^ Given the increasing incidence of CRC, an improved understanding of the disease characteristics and clinical practices in the Middle East and North Africa (MENA) is needed.

Mutations in rat sarcoma virus *(RAS)* proteins are estimated to occur in nearly 25% of all cancers worldwide.^6^ Kirsten-*RAS* (K-Ras), Harvey-*RAS* (H-Ras), and neuroblastoma-*RAS* (N-Ras) proteins are small guanosine triphosphatases that regulate intracellular signaling mediated by the Ras-rapidly accelerated fibrosarcoma (Raf)-mitogen-activated protein kinase kinase (MEK)-extracellular-signal-regulated kinase (ERK) [Ras-Rad-MEK-ERK] and phosphatidylinositol-3-kinase (PI3K)-protein kinase B (Akt)-mammalian target of rapamycin (mTOR) [PI3K-Akt-mTOR] pathways.^7^ Mutations in the *RAS* proto-oncogenes often lead to constitutive activation of downstream signaling, resulting in aberrant cell growth and proliferation. *RAS* mutations are known drivers of CRC tumorigenesis and are estimated to occur in approximately 35%-45% of cases worldwide.^8,9^ Knowledge of *RAS* mutation status plays an important role in clinical decision-making, as the presence of *RAS *mutations has been shown to predict resistance to anti-epidermal growth factor receptor (anti-EGFR) therapies such as cetuximab (Erbitux; Merck KGaA, Darmstadt, Germany) and panitumumab (Vectibix; Amgen, Thousand Oaks, Calif, USA).^10,11^ Within the Middle East region, a meta-analysis of real-world data has estimated a *RAS* mutation prevalence of 33.7%-34.6%.^12^ However, data regarding the frequency of *RAS* mutations in Middle Eastern and African patients with CRC and the impact of *RAS* mutation testing on therapeutic approaches remain limited. To further understand *RAS* testing practices and mutation status in new or recently diagnosed patients with metastatic CRC (mCRC) in the MENA region, we report here the results of a retrospective and prospective evaluation performed across 5 centers in this region.

## Materials and Methods

**Figure 1.**
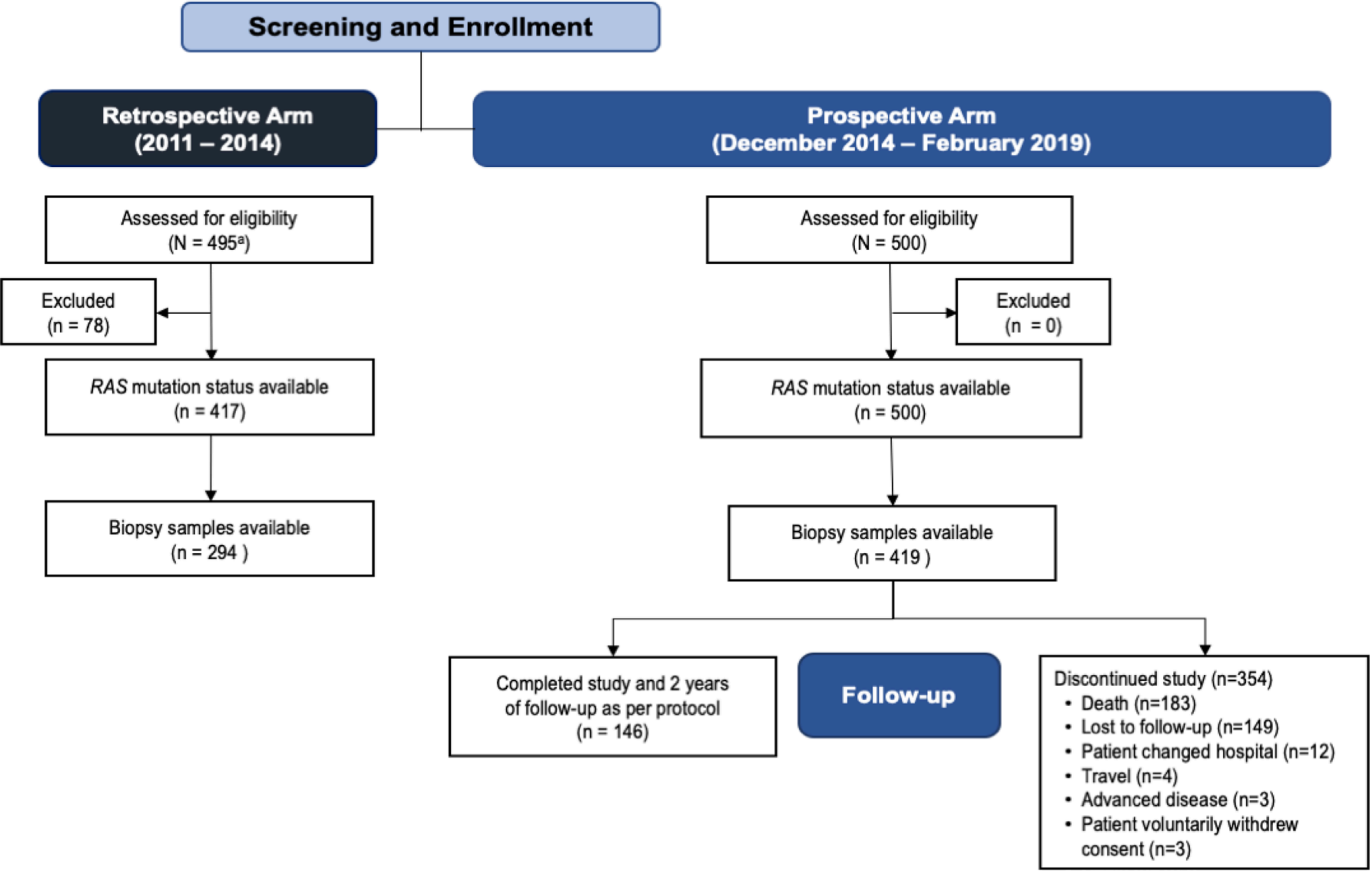
Study enrollment and completion. ^*^Three patients failed to provide informed consent. RAS, rat sarcoma virus.

This was a multi-center, multi-country, observational ambispective study, containing retrospective and prospective arms (Figure 1). Each arm included approximately 500 patients with mCRC across 5 countries in the MENA region, including Algeria, Egypt, the Kingdom of Saudi Arabia (KSA), Kuwait, and Lebanon. The study was approved by the ethics committees of Beni Messous Hospital (Algeria), Alexandria University Faculty of Medicine, Cairo University Faculty of Medicine, National Cancer Institute and Central Ethics Committee of the Egyptian Ministry of Health (Egypt), Kuwait Ministry of Health (Kuwait), King Fahd Medical City IRB, King Saud University IRB and King Abdullah International Medical Research Center (KAIMRC) (KSA), Hammoud Hospital University Medical Center IRB and Hotel Dieu de France EC (Lebanon); and informed consent was obtained from patients prior to study initiation.

For the retrospective arm, patient medical records were analyzed 2 years prior to the study start. Patients aged >18 years with mCRC who had initiated first-line therapy in 2011 or later but prior to study initiation (2014), who had a baseline visit and at least 1 additional visit for disease evaluation, and for whom survival data were available were eligible for inclusion. Patients with incomplete or unavailable data or the presence of other coexisting malignancies were excluded.

For the prospective arm of the study, patients were enrolled between December 2014 and May 2019 and followed for 2 years after enrollment. Patients with newly diagnosed mCRC with at least 1 measurable lesion according to modified Response Evaluation Criteria In Solid Tumors criteria^13^ and availability of paraffin-embedded tumor tissue from the primary tumor or metastasis for biomarker analysis were included in the study. Exclusion criteria included pregnancy or lactation, presence of a co-existing malignancy, life expectancy <6 months, presence of any co-existing medical or psychological condition that would compromise the ability to provide data disclosure authorization, or any other significant disease that could influence the study results, as determined by the investigator.

Considering the overall goal of evaluating past and current practices of *RAS* testing for patients with newly diagnosed mCRC, information related to *RAS* testing was recorded, including the status of test prescription, treatment stage at the time of prescription, reason for non-prescription, method of sample excision, and testing technique. Additionally, information regarding patient characteristics and clinical variables, including gender, tumor stage and Eastern Cooperative Oncology Group classification,^14^ primary tumor site, metastases, initial therapy, response to treatment, changes in therapy, *RAS* testing methods, response to the European Organisation for Research and Treatment of Cancer quality of life questionnaire (EORTC QLQ-C30 Version 3.0), and survival status, was recorded for each patient.

### Statistical Analysis

No formal statistical hypothesis was evaluated, and a descriptive analysis was performed. Cross-tabulations were performed to evaluate the relationship between *RAS* testing prescription, and impact of *RAS* status on therapeutic strategy, and other variables of interest. Logistic regression analyses were performed to identify factors that could promote the prescription of *RAS* testing. Statistical analyses were performed using SAS software version 9.2 (SAS Institute Inc. Cary, NC, USA).

## Results

### Patient Characteristics

Overall, 495 (mean age, 56.1 years) and 500 (mean age, 54.5 years) patients were enrolled in the retrospective arm and prospective arms, respectively (Figure 1). Of these, 487 and 500 cases, respectively, provided evaluable demographic data (Table 1) and *RAS *testing status was available for 417 patients and 500 patients, respectively. All patients in both arms had stage 4 disease with the most frequent primary tumor site in the sigmoidal colon or rectum. In nearly all cases (>96%), the tumor histology was identified as adenocarcinoma. The geographic distribution from the retrospective (211 [42.6%]) as well as prospective arm is reported in Table 1.

Rat Sarcoma Virus Testing: Rates of Prescription and Influencing Factors

Rat sarcoma virus testing was ordered for 326/417 patients in the retrospective arm and 407/500 in the prospective arm of the study (Table 2). Logistic regression analyses indicated that lack of test availability and financial reasons were 2 of the major causes of non-prescription of *RAS* testing (Table 2). These data were corroborated in both arms of the study. Medical decisions and “other reasons” were additional drivers cited for non-prescription of *RAS* mutation testing. Note that for this analysis, only cases in which either *RAS* testing was prescribed or not prescribed due to medical decisions or “other reasons” were considered.

In the retrospective study arm, age, gender, and duration of condition did not significantly impact the prescription of *RAS* testing. Correlation analyses between *RAS* testing and primary tumor sites, types, and grades indicated that *RAS* testing prescription did not significantly differ by primary tumor site (*P *= .371) or tumor histology (*P *= .092), although patients with adenocarcinoma were more likely to be prescribed *RAS* testing (Table 3).

In the prospective arm of the study, older age significantly predicted prescription of *RAS* testing (*P* = .028). Additionally, primary tumor site in the ascending versus descending colon (odds ratio [OR] = 0.157, 95% CI = 0.049-0.501, *P* = .002); transverse colon (OR = 0.158, 95% CI = 0.043-0.580, *P* = .005); sigmoidal colon (OR = 0.055, 95% CI = 0.011-0.264, *P* < .001); and rectum (OR = 0.214, 95% CI = 0.075-0.609, *P* = .004) showed significant difference in rate of testing prescription. There was also a correlation of tumor grade with *RAS* testing prescription, with patients having grade 1 (G1) tumors receiving significantly less prescriptions than those having G2 (OR = 0.311, 95% CI = 0.098-0.990, *P* = .048); G3 (OR = 0.151, 95% CI = 0.043-0.529, *P *= .003); and G4 (OR = 0.086, 95% CI = 0.017-0.437, *P *= .003) grade tumors. There was no significant difference in prescription rate between tumors that had an undetermined grade (GX; i.e., grade could not be assessed) and G1 tumors (OR = 3.524, 95% CI = 0.811-15.308, *P *= .093).

The frequency of prescription for *RAS t*esting in each country included in this study was also evaluated in the prospective arm and showed some regional variability, with *RAS* testing prescribed in 91% of the cases in Algeria, 58% in Egypt, 96% in Kuwait, 91% in KSA, and 100% in Lebanon.

### Rat Sarcoma Virus Mutation Frequencies

The frequency of types of *RAS *mutations was evaluated in 271/294 cases and 407/419 cases with available biopsy samples in the retrospective and prospective arms, respectively. The primary tumor was the biopsy source for a majority of cases (retrospective arm: 272 [92.5%]; prospective arm: 365 [87.1%]); the remaining samples were derived from metastases. Overall, more than 90% of samples were provided as paraffin-embedded tissue, and sequencing was the most common method of *RAS* testing, performed in most cases (~78% of cases in both arms). High-resolution melting and allelic discrimination were among the other methods used for *RAS* testing in the remainder of the cases.

**Figure 2.**
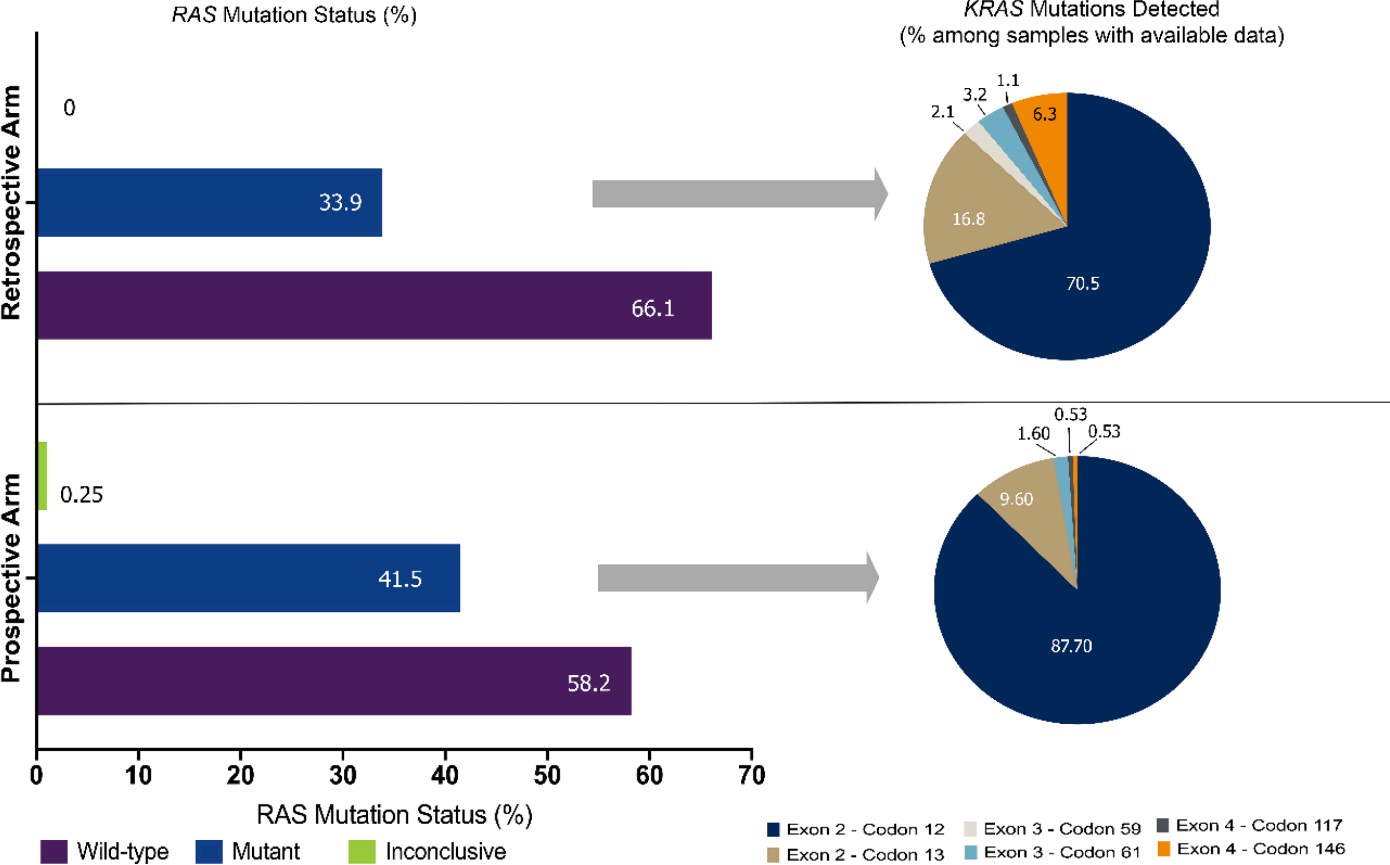
RAS mutation status. KRAS, Kirsten rat sarcoma virus; RAS, rat sarcoma virus.

Among tested samples, *RAS *mutations were detected in 33.9% of cases in the retrospective arm and 41.5% of cases in the prospective arm (Figure 2). In the prospective arm, these cases included 346 samples (69%) with *KRAS* mutations, 20 (4%) with *NRAS* mutations, and 41 (8%) where the mutation was not specified. In both arms, the most commonly observed *RAS* mutations occurred in exon 2, codons 12 and 13 (Figure 2). In the prospective arm, samples were also evaluated for mutations in V-raf murine sarcoma viral oncogene homolog B1 (*BRAF*), phosphatase and tensin homolog (*PTEN*), and *PI3KCA*. Mutations were observed in 4 of 92 (0.8%) cases with available *BRAF* data, 3 of 87 (0.6%) cases with *PTEN* data, and 11 of 78 (2.2%) cases with *PI3KCA *data.

In the prospective arm, the association between the primary tumor localization site and *RAS* testing was statistically significant (*P *= .03). Transverse colon as a primary tumor site had the highest percentage of patients testing positive for mutant (MT) *RAS* while rectum had the highest proportion of patients testing positive for wild-type (WT) *RAS. *In general, *RAS* MT was slightly more on the right, and *RAS* WT was higher on the left side.

### Impact of ***RAS*** Testing on Clinical Decision-Making

A secondary objective of this study was to evaluate the impact of *RAS* testing on therapeutic decisions. In the retrospective arm, most patients received surgery and/or chemotherapy as initial therapy prior to enrollment in the study (data not shown). *RAS* testing was significantly more prescribed after initiation of first-line treatment and in patients with stage IV disease (*P *< .005) and resulted in the addition of targeted therapy (41.8% anti-EGFR, 30.2% anti-vascular endothelial growth factor [anti-VEGF]) in WT mCRC. Testing also significantly impacted the treatment strategy of left-sided tumors (*P *= .037).

In the prospective arm of this study, *RAS* testing results impacted therapy selection in 406 cases (Table 4). Overall, knowledge of *RAS* status resulted in the addition of bevacizumab (Avastin; Roche, Basel, Switzerland) or anti-EGFR therapy in 20.4% and 21.2% of patients, respectively, at visit 1 (Tables 4 and 5). Among the 253 patients with *RAS* WT, 96 (37.8%) received cetuximab, 74 (29.1%) received panitumumab, 48 (18.9%) received bevacizumab, 25 (9.8%) received a change in chemotherapy, and 10 (3.9%) received other therapy changes. Among the 153 cases with *RAS* MT, 134 (87.6%) received bevacizumab, 8 (5.2%) received a change in chemotherapy, 1 (0.7%) received panitumumab, and 10 (6.5%) received other therapy changes.

### Quality of Life

The EORTC QLQ-30 questionnaire was administered at each visit to patients in the prospective study. A slight increase in the score was observed with time, indicating a slight decrease in the quality of life of participants from visit 1 to visit 5 (mean score [standard deviation]: 66 [14] to 70 [22]).

## Discussion

To our knowledge, this is the first large study conducted in the MENA region to evaluate *RAS* testing and its impact on treatment strategies in patients with newly diagnosed mCRC. Our results indicate that although testing was typically prescribed after initiation of first-line treatment in the retrospective arm, most patients in both arms of the study (retrospective: 78%, prospective: 81.4%) were tested for *RAS *mutations. These results are like those from other regions of the world.^15,16^ In 2016, a European survey conducted in 24 countries, involving 96 laboratories, evaluated the implementation of *RAS* testing methods.^17^ Similar to our study, the majority of the participating laboratories (72.9%) in that study reported that testing of all required *RAS* codons was standard practice.^17^ Since the introduction of anti-EGFR therapies in 2004 (cetuximab) and 2006 (panitumumab) and the requirement to assess *RAS* status prior to treatment initiation, *RAS* testing has been increasingly accepted and implemented worldwide.^18^ Indeed, a study from the United States indicated that the annual testing for *KRAS *mutations in newly diagnosed CRC patients increased from 27.4% to 78.4% from 2008 to 2011.^19^

Reasons for not employing *RAS* testing are many but largely changeable. In our study, the observed reasons included financial reasons, medical decision, and unavailability of the tests. Due to the significance of *RAS* mutation results in therapeutic decision-making, efforts should be made to more widely incorporate this test in centers that manage patients with CRC. As well, improvements in reimbursement programs will help facilitate patient access to *RAS* testing.

Our retrospective analysis did not identify factors that significantly impacted the prescription of *RAS* mutation testing. However, the prospective analysis revealed that age, primary tumor site, and tumor grade highly influenced *RAS* testing prescription. Previous studies have assessed potential factors influencing *KRAS* testing prescription. In a study of molecular testing prescribing patterns in France, *KRAS* testing was requested by only 65.5% of treating physicians, and reported factors influencing its prescription included patient age at diagnosis, primary tumor site, and stage at diagnosis.^16^ Geographical location and the general status of the treating center also impacted *KRAS *testing. A study of *KRAS* testing rates in community-based oncology settings in the United States found that elderly age, clinical trial enrollment, financial status, performance status, and presence of comorbid conditions influence *KRAS* mutation testing.^19^ Influencing factors can be classified as (1) disease-related, (2) financially related, (3) patient-related, or (4) treatment-center related. Therefore, further large multi-centric studies are needed to properly assess the influencing factors that impact physician request for *RAS* mutation testing so that optimal testing and evidence-based treatment decisions can be facilitated.

Most tumors in both the retrospective (40.9%) and prospective (33.4%) arms in our study arose from the sigmoid colon. Although the tumor site did not affect the overall rate of prescription of *RAS* testing in the retrospective arm, when available, test results significantly impacted treatment strategy for left-sided tumors. In the prospective arm, the ascending colon (including caecum) as primary tumor site was a key driver of *RAS* testing prescription.

Our study also found a significant relationship between the primary tumor site and *RAS *mutation status. Patients with tumors arising from the right colon had slightly increased incidence of *RAS* mutations similar to the findings of a European study that found a significantly higher prevalence of *RAS* mutations in right versus left-sided tumors.^17^

Overall, *RAS* mutations were detected in approximately the same percentage of patients in both arms of the study (~33.8%). In previous analyses, *RAS* mutation prevalence has been reported to be higher (43%-55.9%) than in our study.^12,17,20^ This discrepancy could be related to the different techniques used to determine *RAS* status, variable percentages of neoplastic cells present in the acquired sample, differences between laboratories, absence of external quality assurance programs, availability of next-generation sequencing, criteria for patient selection, and data analysis methods.^12,17,21^ These factors may also contribute to the geographic variance in *RAS *mutation rates with the highest prevalence of mutant *RAS* in KSA and Kuwait and lowest in Lebanon and Egypt. The sample size of enrolled patients from each country may also contribute to the variation. A multinational survey in 2016 found no significant difference in the prevalence of *RAS* mutations by country (range, 40%-52.1%; *P *= .461).^17^

In recent years, detection of *KRAS* mutations has become necessary to appropriately manage patients with mCRC, especially when considering first-line anti-EGFR monoclonal antibody therapies. *KRAS* mutations have been reported in up to 35%-45% of CRC, with the majority of mutations occurring in exon 2 codon 12.^22,23^ In the retrospective arm of our study, the prevalence of *KRAS* mutations was consistent with previous randomized trials. In the prospective arm of the study,* BRAF* mutations were reported in 0.8% of cases with available *BRAF *data. Testing for microsatellite instability and human epidermal growth factor receptor 2 status are the focus of recent publications^24,25^ and were not obvious targets at the time of our study.

Rat sarcoma virus mutation status is an important element in the decision to prescribe monoclonal antibody therapy. Colorectal cancer patients with *KRAS* mutations, specifically exon 2 codons 12 and 13, do not respond to anti-EGFR therapies such as cetuximab or panitumumab alone or in combination with oxaliplatin (Eloxatin; Sanofi-Aventis, Gentilly, France)-based chemotherapy and are believed to have poor survival.^23,26-28^ *KRAS* exon 2 mutation testing is recommended to predict the benefit from anti-EGFR therapies.^29^

In our study, the addition of bevacizumab to the treatment regimen was the most frequent change in the prospective arm and the second most frequent change in the retrospective arm. This result is reasonable because bevacizumab is the only effective option for tumors with mutant *RAS*.

Tumors with wild-type *RAS* have been shown to benefit from anti-EGFR therapies, including cetuximab and panitumumab.^12,30,31^ In our study, cetuximab was not prescribed in any patients with *RAS *mutations; however, cetuximab was the most commonly prescribed regimen, followed by panitumumab. The changes in treatment regimens after *RAS* testing reflect physician awareness in the Middle East region of the need for *RAS* testing and the implications for patient management.

This study has several limitations, in particular, the data from the retrospective arm of the study are often incomplete. The study population is not representative of the entire MENA region as it is heterogeneously recruited from only 5 countries in the region. The impact of RAS testing on the choice of treatment is not always obvious due to the frequent delays in receiving results.

## Conclusions

In conclusion, our study indicates that although *RAS *testing previously appeared to be prescribed later in the management of patients with CRC, testing is now a common practice in managing newly diagnosed patients with mCRC in the MENA region. However, there is room for improvement. Reasons for not prescribing *RAS* testing were clear and must be addressed to improve future outcomes. The primary tumor site influenced the rate of *RAS* testing, mutation prevalence, and management decisions. Together, the assessment of *RAS* mutations represents an important element in clinical decision-making for mCRC, and *RAS *testing can significantly impact treatment decisions.

## Data Availability:

Qualified researchers may request data from Amgen clinical studies. Complete details are available at the following: http://www.amgen.com/datasharing.

## Figures and Tables

**Table 1.** Demographic Characteristics

	**Retrospective, n (%) **	**Prospective, n (%)**
**Gender**	**(n = 487)**	**(n = 500)**
Male	271 (55.6)	210 (42)
Female	216 (44.4)	290 (58)
**Country**	(n = 495)	(n = 500)
Algeria	211 (42.6)	248 (49.6)
Egypt	110 (22.2)	157 (31.4)
KSA	62 (12.5)	32 (6.4)
Kuwait	50 (10.1)	50 (10)
Lebanon	23 (4.6)	13 (2.6)
UAE	39 (7.9)	0 (0)
**Body mass index, mean kg/m^2^ (SD)**	**(n = 417)**	**(n = 493)**
**Smoker**	22.31 (9.0)	25.39 (5.29)
Yes	81 (16.7)	96 (19.2)
No	405 (83.3)	404 (80.8)
**Family history of cancer, n (%)**	**(n = 484)**	**(n = 498)**
Yes	96 (19.8)	114 (22.8)
No	388 (80.2)	384 (76.8)
First degree	69/96 (71.9)	84/113 (74.3)
Second degree	23/96 (24.0)	29/113 (25.7)
First and second degree	2/96 (2.1)	0/113 (0)
Data not available	2/96 (2.1)	0/113 (0)
**Primary tumor site**	**(n = 448)**	**(n = 500)**
Ascending colon	77 (17.2)	92 (18.4)
Descending colon	54 (12.0)	57 (11.4)
Rectum	117 (26.1)	148 (29.6)
Sigmoid	183 (40.9)	167 (33.4)
Transverse colon	17 (3.8)	26 (5.2)
Unknown	0 (0.0)	7 (1.4)
Missing	0 (0.0)	3 (0.6)
**Tumor grade**	**(n = 448)**	**(n = 500)**
GX	154 (34.4)	172 (34.4)
G1	66 (14.7)	59 (11.8)
G2	144 (32.1)	166 (33.2)
G3	52 (11.6)	76 (15.2)
G4	16 (3.6)	20 (4.0)
Unknown	16 (3.6)	4 (0.8)
Missing	0 (0.0)	3 (0.6)
**ECOG performance score**	**(n = 447)**	**(n = 500)**
0	192 (43.0)	184 (36.8)
1	207 (46.2)	232 (46.4)
2	43 (9.7)	71 (14.2)
3	2 (0.52)	4 (0.8)
4	1 (0.26)	1 (0.2)
5	1 (0.26)	0 (0)
Missing data	0 (0)	8 (1.6)

ECOG, Eastern Cooperative Oncology Group; G, tumor grade; GX, tumor grade undetermined (i.e., grade could not be assessed); KSA, Kingdom of Saudi Arabia; SD, standard deviation; UAE, United Arab Emirates.

**Table 2.** Rates of RAS Mutation Testing Prescriptions

	**Retrospective, n (%)**	**Prospective, n (%)**
RAS mutation testing prescribed	(n = 417)	(n = 500)
Yes	326 (78.2)	407 (81.4)
No	91 (21.8)	93 (18.6)
Reasons for non-prescription of RAS mutation testing	(n = 91)	(n = 93)
Financial	19 (20.9)	23 (24.7)
Medical decision	15 (16.5)	19 (20.4)
Unavailability	34 (37.3)	51 (54.8)
Others	23 (25.3)	0 (0)

RAS, rat sarcoma virus.

**Table 3.** Correlation Between RAS Testing Prescription and Tumor Characteristics: Retrospective Arm

	RAS **Prescription (n)**	
	**Yes**	**No**
**Tumor site (n = 393) (** ***P*** ** = .371)**		
Ascending colon	53	11
Descending colon	36	11
Rectal	79	29
Sigmoid	133	28
Transverse colon	10	3
**Tumor histology (n = 408) (** ***P*** ** = .092)**		
Adenocarcinoma	316	86
Other	3	3
**Tumor grade (n = 396) (** ***P*** = .025 ^*^ )		
G1	46	17
G2	94	40
G3	29	9
G4	7	1
GX	131	22

^*^ *P*-value significant at <.05.

G, tumor grade; GX, tumor grade undetermined (i.e., grade could not be assessed); RAS, rat sarcoma virus.

**Table 4.** Changes to Therapy as a Result of RAS Testing

	**Retrospective (n = 225)**	**Prospective (n = 406)**	
	**All Cases**	**Mutant Type (N = 153)**	**Wild Type (N = 253)**
Added bevacizumab	68 (30.2%)	134 (87.6%)	48 (18.9%)
Added cetuximab	94 (41.8%)	0 (0.0%)	96 (37.8%)
Added panitumumab	19 (8.4%)	1 (0.7%)	74 (29.1%)
Change in chemotherapy	10 (4.5%)	8 (5.2%)	25 (9.8%)
Other	34 (15.1%)	10 (6.5%)	10 (3.9%)

RAS, rat sarcoma virus.

**Table 5.** Correlation Between Tumor Characteristics and Impact of RAS Status on Therapeutic Strategy

	**RAS Testing Effect on Therapy (n)**				
	Added Bevacizumab	Added Cetuximab	Added Panitumumab	Change in Chemotherapy	Other
**Retrospective**						**Tumor site (n = 204) (** ***P*** = .037 ^*^ )					
Ascending colon	13	12	5	2	2
Descending colon	2	12	3	1	7
Rectal	21	23	0	0	6
Sigmoid	20	39	9	7	17
Transverse colon	1	2	0	0	0
**Tumor Histology (n = 208) (** ***P *** **= .137)**					
Adenocarcinoma	60	88	17	9	31
Other	0	1	0	1	1
**Prospective**					
**Visit 1 (** ***P*** ** = .5)**					
Ascending colon	19 (50.0%)	10 (26.3%)	5 (13.2%)	3 (7.9%)	1 (2.6%)
Descending colon	11 (45.8%)	4 (16.7%)	6 (25%)	2 (8.3%)	1(4.2%)
Rectal	30 (42.3%)	24 (33.8%)	13 (18.3%)	3 (4.2%)	1 (1.4%)
Sigmoid	32 (42.1%)	21 (27.6%)	19 (25%)	3 (3.9%)	1 (1.3%)
Transverse colon	10 (58.8%)	2 (11.8%)	2 (11.8%)	2 (11.8%)	1 (5.9%)
**Visit 2 (** ***P*** ** = .7)**					
Ascending colon	11 (73%)	1 (7%)	2 (13%)	1 (7%)	0 (0.0%)
Descending colon	2 (29%)	3 (43%)	1 (14%)	1 (14%)	0 (0.0%)
Rectal	8 (40%)	5 (25%)	3 (15%)	3 (15%)	1 (5%)
Sigmoid	12 (43%)	6 (21%)	6 (21%)	2 (7.5%)	2 (7.5%)
Transverse colon	4 (66%)	1 (17%)	0 (0.0%)	0 (0.0%)	1 (17%)

^*^ *P*-value significant at <.05.

RAS, rat sarcoma virus.
